# Supplementary material for: Heparin-based hydrogel scaffolding alters the transcriptomic profile and increases the chemoresistance of MDA-MB-231 triple-negative breast cancer cells
Source: Biomater Sci. 2020 Feb 13;8(10):2786–96. doi: 10.1039/c9bm01481k (PMC7497406; doi:10.1039/c9bm01481k)
Supplement: Supplementary file 2 [file BM-008-C9BM01481K-s002.zip › Supplementary File 4/HP-BhydrogelvControl/Pathways/my_analysis.Gsea.1545201079907/HALLMARK_NOTCH_SIGNALING.html]

Details for gene set HALLMARK\_NOTCH\_SIGNALING[GSEA]

|  || Dataset | expr.class.cls#HYD\_versus\_CONTROL.class.cls#HYD\_versus\_CONTROL\_repos |
| Phenotype | class.cls#HYD\_versus\_CONTROL\_repos |
| Upregulated in class | HYD |
| GeneSet | HALLMARK\_NOTCH\_SIGNALING |
| Enrichment Score (ES) | 0.317969 |
| Normalized Enrichment Score (NES) | 0.9816209 |
| Nominal p-value | 0.4743976 |
| FDR q-value | 0.94170016 |
| FWER p-Value | 1.0 |
Table: GSEA Results Summary

  

Fig 1: Enrichment plot: HALLMARK\_NOTCH\_SIGNALING      
 Profile of the Running ES Score & Positions of GeneSet Members on the Rank Ordered List

  

| PROBE | DESCRIPTION (from dataset) | GENE SYMBOL | GENE\_TITLE | RANK IN GENE LIST | RANK METRIC SCORE | RUNNING ES | CORE ENRICHMENT || 1 | NOTCH1 | na |  |  | 1056 | 1.835 | 0.0540 | Yes |
| 2 | FZD7 | na |  |  | 2039 | 1.347 | 0.0829 | Yes |
| 3 | PPARD | na |  |  | 2220 | 1.265 | 0.1487 | Yes |
| 4 | DTX2 | na |  |  | 2382 | 1.215 | 0.2126 | Yes |
| 5 | HES1 | na |  |  | 3253 | 0.976 | 0.2252 | Yes |
| 6 | KAT2A | na |  |  | 3384 | 0.943 | 0.2745 | Yes |
| 7 | PSEN2 | na |  |  | 3877 | 0.838 | 0.2986 | Yes |
| 8 | PRKCA | na |  |  | 4876 | 0.646 | 0.2850 | Yes |
| 9 | FZD5 | na |  |  | 4966 | 0.633 | 0.3180 | Yes |
| 10 | LFNG | na |  |  | 5702 | 0.525 | 0.3108 | No |
| 11 | ST3GAL6 | na |  |  | 7368 | 0.316 | 0.2428 | No |
| 12 | CCND1 | na |  |  | 7494 | 0.308 | 0.2546 | No |
| 13 | NOTCH2 | na |  |  | 8394 | 0.208 | 0.2201 | No |
| 14 | FZD1 | na |  |  | 8501 | 0.206 | 0.2268 | No |
| 15 | DTX4 | na |  |  | 9560 | 0.093 | 0.1772 | No |
| 16 | TCF7L2 | na |  |  | 9666 | 0.078 | 0.1763 | No |
| 17 | NOTCH3 | na |  |  | 9955 | 0.045 | 0.1640 | No |
| 18 | ARRB1 | na |  |  | 11079 | -0.075 | 0.1099 | No |
| 19 | MAML2 | na |  |  | 12266 | -0.209 | 0.0604 | No |
| 20 | FBXW11 | na |  |  | 12747 | -0.251 | 0.0503 | No |
| 21 | PSENEN | na |  |  | 13378 | -0.334 | 0.0373 | No |
| 22 | RBX1 | na |  |  | 13530 | -0.357 | 0.0507 | No |
| 23 | WNT5A | na |  |  | 13835 | -0.411 | 0.0593 | No |
| 24 | CUL1 | na |  |  | 14693 | -0.522 | 0.0456 | No |
| 25 | SAP30 | na |  |  | 15285 | -0.614 | 0.0513 | No |
| 26 | JAG1 | na |  |  | 15843 | -0.713 | 0.0646 | No |
| 27 | APH1A | na |  |  | 16269 | -0.801 | 0.0901 | No |
| 28 | SKP1 | na |  |  | 17209 | -1.054 | 0.1038 | No |
Table: GSEA details [plain text format]

  

Fig 2: HALLMARK\_NOTCH\_SIGNALING      
 Blue-Pink O' Gram in the Space of the Analyzed GeneSet

  

Fig 3: HALLMARK\_NOTCH\_SIGNALING: Random ES distribution      
 Gene set null distribution of ES for **HALLMARK\_NOTCH\_SIGNALING**
